# Gender and Educational Stage Differences in Motivation, Basic Psychological Needs and Enjoyment: Evidence from Physical Education Classes

**DOI:** 10.3390/children11121503

**Published:** 2024-12-10

**Authors:** Rubén Navarro-Patón, Josune Rodríguez-Negro, María Muíño-Piñeiro, Marcos Mecías-Calvo

**Affiliations:** 1 Facultade de Formación do Profesorado, Universidade de Santiago de Compostela, 27001 Lugo, Spain; ruben.navarro.paton@usc.es (R.N.-P.); maria.muino.pineiro@rai.usc.es (M.M.-P.); marcos.mecias@usc.es (M.M.-C.); 2 Department of Physical Education and Sport, Faculty of Education and Sport, University of the Basque Country (UPV/EHU), 01007 Vitoria-Gasteiz, Spain

**Keywords:** self-determination theory, primary education, secondary education, children, intrinsic motivation, external regulation, amotivation, competence, relatedness, autonomy

## Abstract

Background/Objectives: There is evidence that shows an association between basic psychological needs, motivation, and enjoyment in the context of school physical education. However, there are no studies that have included all of them in a single study. Therefore, the objective of this research was to determine if there are differences in motivation, basic psychological needs, and enjoyment in physical education classes between primary and secondary education students and between boys and girls. Methods: A total of 740 schoolchildren (410 girls, 55.4%) from primary education (n = 310; 41.9%) and secondary education (n = 430; 58.1%) between the ages of 10 and 17 participated (M = 13.60; SD = 2.03). The Perceived Locus of Causality in Physical Education Scale (PLOC Scale), the Basic Psychological Needs in Exercise Scale (BPNES), and the Physical Activity Enjoyment Measurement Scale (PACES) were administered. Results: Statistically significant differences were found in the educational stage factor with higher scores in primary education students in intrinsic regulation (*p <* 0.001), identified regulation (*p <* 0.001), introjected regulation (*p =* 0.004), perceived competence (*p <* 0.001), relatedness (*p <* 0.001), and enjoyment (*p <* 0.001). In terms of the gender factor, there were significant differences in intrinsic regulation (*p =* 0.005), identified regulation (*p =* 0.007), and enjoyment (*p =* 0.010), with higher scores in boys. Conclusions: Primary education students present greater self-determined motivation (intrinsic, identified, and introjected regulation), as well as a greater sense of competition, relatedness, and enjoyment than secondary education students. Boys have greater regulation and intrinsic enjoyment than girls in physical education classes.

## 1. Introduction

The human being, by definition, is an active being, constantly adopting a wide variety of behaviors to survive [1], adapting to the environment, using both internal and external means, and responding to changes and needs as they arise [2].

The thinking, behavior, and way of acting of students in the field of physical education (PE) continue to be the object of study due to the implications they have [3], in addition to the information that helps teachers understand how motivation, enjoyment, or the feeling of competence combine and interact to impact student learning [4]. Therefore, it must be taken into account that, in childhood and adolescence, school PE is an ideal element to improve participation in physical activities since almost 100 percent of schoolchildren must go to school [5,6]. On the other hand, the key elements in this participation in physical activities are enjoyment [7,8], motivation [9,10], and the satisfaction of basic psychological needs (i.e., autonomy, competence, and relatedness [11] as drivers of participation in extracurricular physical activity [9,12]).

One of the most widely used theories for the study of motivation, the satisfaction of basic psychological needs, and enjoyment in PE is self-determination theory (SDT; [13]), understood as a broad framework for the explanation of motivation and human behavior [14]. SDT is made up of several mini theories, including the theory of basic psychological needs. These needs refer to the psychological construct that people need to feel motivated to behave and determine the existence of at least three basic psychological needs that explain human behavior: the need for autonomy, the need for competence, and the need for relatedness [14,15]. The first, autonomy, refers to the need that people have to have or feel the ability to make decisions for themselves in various life activities. If this ability is frustrated, passive and dependent behaviors may appear, as well as emotional futility and hopelessness [13,14,15]. The need for competence, and therefore to perceive one’s own capabilities, is fundamentally related to the previous one in the sense that it is based on the ability to control what happens with one’s own actions, but in this case it focusses on the belief that one has sufficient resources to carry out certain behaviors. It is the belief that we are capable and that the actions we choose to perform autonomously can be carried out successfully thanks to our capabilities and that they will, to a certain extent, influence what happens [15]. Finally, the need for relatedness has to do with the feeling or need to feel part of a group with which we can interact positively and form mutually supportive relatedness [16]. Satisfying these needs provides energy to perform certain behaviors, such as physical activity [13]. Previous research shows that meeting students’ psychological needs has a positive effect on their enjoyment of PE [8,10]. In addition, different research has found differences in the satisfaction of these needs by gender [17].

Following the mini theory of causal orientations, the existence of three main types of motivation is established: intrinsic or autonomous, extrinsic or controlled, and impersonal or unmotivated [13,14]. In the case of intrinsic or autonomous motivation, it represents what motivates us to act from within and carry out a behavior because it is fun [14,18], since the three basic psychological needs are satisfied, which leads the individual to act based on their will and choice [15]. On the other hand, there are more extrinsic forms of motivation that can be divided into three types of controlled or extrinsic regulation [14]: external regulation, in which activities are carried out due to external stimuli or factors, such as to obtain a reward or, in the case of physical education, a good grade [18,19,20]; introjected regulation, in which activities are carried out due to internal pressures or thoughts (for example, the feeling of guilt); and identified regulation, in which the person acts due to the benefits that arise from carrying out the activity [18,19,20,21]. Furthermore, it is also necessary to include integrated regulation, which, according to self-determination theory (SDT), represents the highest level of the internalization of extrinsic motivation. In this type of regulation, people perform activities because they have fully integrated them into their personal identity and values [22]. For example, a student may participate in physical education classes not only because they recognize their benefits but also because physical activity has become an essential part of their healthy lifestyle. Finally, amotivation is derived from the feeling of a lack of competence and autonomy, where the subject thinks that actions have no effect on reality, has no interest, or feels incapable of carrying out an activity [13]. Previous research has shown that these different types of motivational regulation are associated with emotional consequences such as enjoyment [23]. Enjoyment is positively related to autonomous forms of motivation (intrinsic motivation) and negatively related to controlled forms of motivation [10]. These differences also hold with respect to gender [24]. Compared to girls, boys tend to show greater intrinsic motivation and identify and internalize rules to participate in physical education classes for fun and personal satisfaction [25,26]. In terms of basic psychological need satisfaction, boys reported being more satisfied with their needs for competence, autonomy, and relatedness, which translated into greater motivation for and enjoyment of physical activity [27]. In comparison, girls tend to have lower levels of self-determined motivation, enjoyment, and satisfaction of these needs, which may negatively affect their experience in physical education classes [24,28,29].

Given the importance of a relationship between these constructs in the field of school PE and the practice of physical activity with learning, and given the current lack of scientific evidence on this matter, in our country, that combines these elements in primary and secondary education, the objective of this study was to determine whether there are differences in motivation, basic psychological needs, and enjoyment in PE classes between primary and secondary education students and between boys and girls, having as a starting hypothesis that the determined intrinsic motivation plus self-motivation, the satisfaction of basic psychological needs and enjoyment, will be greater in primary education students than in secondary education students and, in turn, will be greater in boys than in girls.

## 2. Materials and Methods

### 2.1. Design

This study was carried out using a cross-sectional observational design and correlation using a quantitative methodology [30].

### 2.2. Participants

The participants in this study belong to 8 primary and secondary education centers in the provinces of A Coruña, Lugo, and Pontevedra (Galicia, Spain), which were selected through non-probabilistic and convenience sampling based on the accessibility of the educational centers and the geographic proximity for researchers.

### 2.3. Instruments

The following instruments were used to collect data:

The Perceived Locus of Causality in EF Scale (PLOC Scale) was used [31]. A Spanish adaptation of the Motivation Scale towards PE Classes by Goudas, Biddle, and Fox [32]. It is made up of 12 items preceded by the statement “I participate in this PE class…”. The items are on a Likert-type scale (where 1 means “totally disagree”; 2 “somewhat disagree”; 3 equals “somewhat disagree”; 4 means “neutral”; 5 “somewhat agree”; 6 “quite agree”; and, finally, 7 “totally agree”). The scale consists of the following five factors: intrinsic motivation (for example, “because PE is fun”), identified regulation (“because I want to learn sports skills”), introjected regulation (“because I want the teacher to think that I am a good student”), external regulation (“because that’s what I’m supposed to do”), and amotivation (“but I don’t understand why we should have PE”).

The Basic Psychological Needs in Exercise Scale (BPNES). An adaptation to PE of the scale of basic psychological needs in exercise by Vlachopoulos and Michailidou [33]; for the Spanish context, a version by Menéndez and Fernández-Río [34] was used. The scale consists of 12 items headed with the phrase “During my Physical Education classes…”. The items correspond to a Likert-type scale, from 1 (totally disagree) to 5 (totally agree). This scale has three factors: autonomy (for example, “the way I do the exercises matches perfectly with the way I want to do them”), competence (for example, “I feel that I have made a lot of progress with respect to the final result that I have proposed”), and relatedness with others (for example, “I feel very comfortable when I exercise with my other colleagues”).

The Physical Activity Enjoyment Measurement Scale (PACES). The adaptation to physical education of the Physical Activity Enjoyment Scale (PACES) by Motl et al. [35] was used for the Spanish context [36]. The scale is composed of a total of 16 items headed by the statement, “When I am active”. The items correspond to a Likert-type scale, ranging from 1 (totally disagree) to 5 (totally agree). It consists of a single factor: enjoyment (e.g., “I feel good”).

### 2.4. Procedure

To carry out the study, the principals and physical education teachers of the participating schools were contacted, providing them with detailed information about the study and requesting their collaboration. Once the authorization of the schools was obtained, permission was requested from the parents and/or legal guardians of the students. Only students with written authorization from their legal guardians participated in the study. The instruments were applied by the researchers during a physical education session in the middle of the school year in paper format. To avoid interference in the students’ responses, the teacher was asked to be absent.

After offering a brief initial explanation and resolving any existing doubts, 15 min were given for each questionnaire, with a 5 min break between each one.

### 2.5. Statistical Analysis

Data analysis for this research was performed using SPSS 28.0 statistical software (SPSS v.28, IBM Corporation, New York, NY, USA) for all analyses. The level of statistical significance was *p <* 0.05.

The descriptive statistics of the variables studied were used to summarize the data. McDonald’s omega was used to study the reliability of the instruments used. The Kolmogorov–Smirnov statistical test was used to determine the normality of the data. The analysis of the relationships between the study variables was determined using the Pearson correlation coefficient. Subsequently, a multivariate analysis (MANOVA) was performed for each variable studied (i.e., intrinsic regulation, identified regulation, introjected regulation, external regulation, amotivation, autonomy, competence, affinity, and enjoyment). Educational stage (primary versus secondary education) and gender (boy versus girl) were included as study factors. The Bonferroni statistic was used to analyze the main effects and the interactions between them. Statistical power was expressed using the eta squared statistic (η^2^). The effect size was considered small (0.01), medium (0.06), or large (0.14 or more) [37].

### 2.6. Ethics

The study has followed the procedures of the Declaration of Helsinki [38] and was approved by the EDUCA Ethics Committee with the code number 10/2024.

## 3. Results

A total of 740 primary school students (n = 310; 41.9%) and secondary school students (n = 430; 58.1%) between the ages of 10 and 17 participated in this study (M = 13.60; SD = 2.03). Of the total sample, 410 were girls (55.4%) and 330 were boys (44.6%).

### 3.1. Reliability, Correlations, and Descriptive Statistics

The reliability analysis showed adequate results for the variables studied; thus, the reliability coefficients (McDonald’s Omega) were as follows: intrinsic regulation, 0.82; identified regulation, 0.81; introjected regulation, 0.69; external regulation, 0.72; amotivation, 0.71; autonomy, 0.70; competence, 0.70; relatedness, 0.81; and enjoyment, 0.92.

The associations between the study variables showed that enjoyment was positively correlated with intrinsic regulation, identified regulation, introjected regulation, autonomy, competence, and relatedness among primary education students (Table 1). There was also a positive correlation between enjoyment and intrinsic regulation and identified regulation. A negative correlation was found between enjoyment and external regulation and amotivation.

Among secondary education students (Table 2), there were positive correlations between intrinsic regulation and identified regulation, introjected regulation, autonomy, competence, relatedness, and enjoyment, and negative correlations with external regulation and amotivation. Enjoyment was positively correlated with intrinsic regulation, identified regulation, introjected regulation, autonomy, and competence. Amotivation and external regulation were negatively correlated with enjoyment.

The descriptive statistics showed that primary education students have higher scores than secondary students in intrinsic regulation, identified and introjected regulation, autonomy, competence, relatedness, and enjoyment (Table 3). However, secondary education students show higher scores in external regulation and amotivation, although the latter is much lower than the scores that appear in the other dimensions.

### 3.2. Multivariate Analysis Based on Gender and Educational Stage

The results of the MANOVA based on the educational stage factor indicate that there are statistically significant differences in intrinsic regulation [F (1, 736) = 61.505, *p <* 0.001, η^2^ = 0.077]; identified regulation [F (1, 736) = 42.753, *p <* 0.001, η^2^ = 0.055]; introjected regulation [F (1, 736) = 8.396, *p =* 0.004, η^2^ = 0.011]; perceived competence [F (1, 736) = 51.720, *p <* 0.001, η^2^ = 0.065]; relatedness [F (1, 736) = 476.531, *p <* 0.001, η^2^ = 0.392]; and enjoyment [F (1, 736) = 52.509, *p <* 0.001, η^2^ = 0.066]. In all the variables studied, primary education students obtained higher scores than secondary education students. No significant differences were found in external regulation (*p =* 0.285), amotivation (*p =* 0.076), or autonomy (*p =* 0.102).

The results of the MANOVA depending on the gender factor indicate that there are statistically significant differences in intrinsic regulation [F (1, 736) = 7.994, *p =* 0.005, η^2^ = 0.011]; identified regulation [F (1, 736) = 7.216, *p =* 0.007, η^2^ = 0.055]; and enjoyment [F (1, 736) = 6.670, *p =* 0.010, η^2^ = 0.009]. In these three variables studied, boys obtain higher scores than girls. No significant differences were found in the rest of the variables (*p >* 0.05).

In pairwise comparisons, significant differences were found in intrinsic regulation (*p =* 0.001), identified regulation (*p =* 0.006), and enjoyment (*p =* 0.040) between secondary school boys and girls with higher scores in boys. Furthermore, significant differences have been found in intrinsic motivation (*p <* 0.001), identified regulation (*p <* 0.001), competence (*p <* 0.001), relatedness (*p <* 0.001), and enjoyment (*p <* 0.001) between children in primary education and secondary education, with higher scores in children in primary education.

Significant differences were found in intrinsic regulation (*p <* 0.001), identified regulation, introjected regulation (*p* = 0.010), competition (*p <* 0.001), relatedness (*p <* 0.001), and enjoyment (*p <* 0.001), among girls of both stages, with higher scores in primary school girls in all the variables studied.

## 4. Discussion

The objective of this study was to determine whether there are differences in motivation, basic psychological needs, and enjoyment in PE classes between primary and secondary education students and between boys and girls. Based on the results obtained, in general, primary education students have a higher intrinsic motivation, an important factor according to the SDT [14,39,40], which maintains that in order to improve intrinsic motivation, the satisfaction of psychological needs is necessary [41]. Furthermore, in general, primary education students obtain higher scores in two of the three basic psychological needs: competence and relatedness, as well as enjoyment. These results are in line with the theoretical assumptions described in the SDT [13,14], which indicates that higher scores in basic psychological needs would directly and indirectly imply greater enjoyment in PE classes [42]. Regarding the comparison between boys and girls, boys present greater intrinsic and identified motivation and greater enjoyment than girls, both in primary and secondary education, as occurred in previous research that demonstrated differences between boys and girls in terms of enjoyment of PE [8,17] and motivational regulation [7,43].

Regarding motivation, previous research has shown that motivation towards PE classes varies within age groups, especially between primary and secondary education students [12,44], as demonstrated by the results of our study. In younger children, there is a higher score in intrinsic, identified, and introjected motivation towards physical education, which would mean that these students have a more self-determined profile [45,46,47]. This may indicate the interest of primary education students in the subject, in which, despite it being mandatory in the educational curriculum and therefore subject to normative evaluation [44], participation can be associated with the pleasure of participating in classes and with fun and enjoyment [48], as demonstrated by the results. Regarding external regulation and demotivation, the scores are higher among secondary education students [12]. These scores are in line with the study by Jaitner et al. [49], and this may be because students must participate in PE sessions in a mandatory manner, either for fear of punishment or for fear of failing the subject (external regulation), or simply because students believe that it is what they should do and it is the right thing to do, and that is why they are forced to participate in classes [50].

In relation specifically to boys, it was found that they presented a higher intrinsic and identified motivation than girls in both primary and secondary education [51], which is consistent with the scientific evidence that indicates that girls present a higher demotivation towards PE than boys [52], consequently showing higher levels of amotivation compared to boys. Girls also present lower scores in intrinsic motivation and introjected regulation, which could be associated with low perceptions of competence and lower interest in the tasks performed in PE classes [52,53].

Regarding the satisfaction of basic psychological needs, primary education students again present higher scores than secondary education students in competence and relatedness with others [8,17]. This could be due to the fact that primary education teachers foster a more collaborative and supportive environment than PE teachers in secondary education [17] or to the fact that the methodology in primary education tends to be more playful and participatory [51]. On the other hand, as in our study, those students who present higher scores in competence and relatedness also present higher scores in enjoyment. These results agree with previous studies that revealed that the satisfaction of students’ psychological needs has a positive impact on enjoyment in the context of physical education; specifically, enjoyment has been related to perceived competence and social relatedness [26,54].

Regarding gender differences, and contrary to what occurs in our study, scientific evidence suggests that boys show higher levels of satisfaction in terms of competence and relatedness [55]. The results of our study may be motivated by the use of interpersonal methodological styles that focus on gender differences among students and try to address the tastes of each of them [56].

Regarding enjoyment in PE classes, primary education students obtain higher scores than secondary education students [48,51]. This may be due to the fact that the learning environment in primary education is usually more playful and less competitive [51]. In addition, primary education teachers tend to use more participatory and student-centered methodologies, which may increase enjoyment [48], or secondary education students may feel greater pressure to improve their academic performance, which may negatively affect their enjoyment of PE classes [57]. Furthermore, girls, both in primary and secondary education, obtain lower scores than boys. This may be motivated by several factors, including differences in socialization, present from an early age in which boys and girls are socialized in relation to sport differently [17], or having had previous negative experiences in PE classes such as feeling excluded or not receiving the same level of attention and support as boys, which can affect enjoyment [58].

As limitations of this study, we would like to highlight that, as it is a cross-sectional study, causality cannot be inferred from the data and the relationships obtained in each of the constructs analyzed, so future studies on this topic should approach it longitudinally to establish cause and effect. The data from this study correspond to an intentional and convenience sample, which would lead us to suggest that future studies attempt to carry out a representative random sample to improve reality. Furthermore, although there is emerging literature regarding the assessment of frustration and neglect of basic psychological needs in school contexts, these variables were not studied in the present study. Finally, as it is a cross-sectional study, this study only shows the student’s thinking at a given time without taking into account the type of content that was being taught in PE classes, so the data should be taken with caution.

## 5. Conclusions

The results of the study allow us to conclude that primary education students present greater self-determined motivation (intrinsic, identified, and introjected regulation) than secondary education students. Likewise, these same students have a greater sense of competition and relatedness than secondary education students. Finally, primary education students obtain higher scores in enjoyment in PE classes. This suggests that primary education students have a more self-determined motivational profile and greater satisfaction of their basic psychological needs than secondary education students. On the other hand, secondary education students present higher scores in external regulation and amotivation, although these differences are not statistically significant. This indicates a tendency towards less self-determination and greater dependence on external factors for their motivation.

Boys have higher motivation and intrinsic enjoyment than girls; that is, boys engage and participate in PE classes because of the pleasure and satisfaction they receive from their own practice. Furthermore, comparisons also show that boys in secondary education have significantly higher scores in intrinsic regulation, identified regulation, and enjoyment compared to girls. This suggests that boys in secondary education are more motivated by internal reasons and find more enjoyment in PE classes compared to girls in the same educational stage. Identified regulation implies that students see a personal value in the activity and consider it important to them.

## 6. Practical Applications

As practical applications of this study, we would like to highlight and show that self-determination in PE and sport consists of three components: emotions, feelings, and cognition. Therefore, students must perceive the subject of PE as pleasant, satisfying, and useful. Thus, these aspects are influenced by several factors, including relatedness with peers in the classroom, type of course, teaching style, methods, or content. Therefore, the teaching strategies necessary to increase student participation in PE classes must include and offer different methods to promote autonomy, provide feedback on the skills performed, and promote respect and care in the group.

Regarding the different methods, teaching strategies could be proposed that use active and participatory teaching methods; that is, using teaching methods that actively involve students, such as project-based learning or cooperative learning.

In terms of feedback, teachers should aim to provide positive and constructive comments that acknowledge students’ achievements; for example, instead of simply saying “good job”, one could say “You’ve really improved your throwing technique; keep it up”, which helps students feel competent and motivated to keep improving.

Regarding the promotion of respect and care in the group, it is proposed, for example, to include activities that generate a classroom environment where positive relatedness between students is encouraged. For example, organizing team activities that require collaboration and mutual support, such as teamwork, empathy, and conflict resolution through the organization of cooperative tasks and games where students must work together to achieve a common goal; norms of respect and care can also be established within the class group, ensuring that all students feel valued and supported. For example, implementing a “buddy” system where students support each other during different tasks.

## Figures and Tables

**Table 1:** Bivariate correlations between the studied dimensions of the PLOC, BPNES, and PACES of primary education students.

Dimensions	M	SD	A	K	1	2	3	4	5	6	7	8	9
1. Intrinsic regulation	5.83	1.32	−0.186	−0.237	1	0.742 **	0.375 **	−0.117 **	−0.312 **	0.188 **	0.064	0.050	0.524 **
2. Identified regulation	5.79	1.24	0.055	0.166		1	0.403 **	0.002	−0.212 **	0.156 **	0.044	0.035	0.448 **
3. Introjected regulation	4.25	1.43	−0.197	−0.368			1	0.476 **	0.161 **	0.168 **	0.088	0.008	0.145 *
4. External regulation	3.95	1.71	0,041	−0.966				1	0.426 **	−0.021	−0.004	−0.028	−0.256 **
5. Amotivation	2.36	1.56	0.071	0.136					1	−0.081	−0.016	−0.044	−0.238 *
6. Autonomy	3.52	0.90	−0.101	−0.278						1	0.448 **	0.293 **	0.228 **
7. Competence	4.13	0.71	−0.072	−0.346							1	0.431 **	0.193 **
8. Relatedness	4.40	0.77	0.234	0.167								1	0.165 **
9. Enjoyment	4.36	0.70	0.78	0.24									1

Note: M: mean; SD: standard deviation; A: asymmetry; K: kurtosis; ** The correlation is significant at the 0.001 level (bilateral). * The correlation is significant at the 0.05 level (bilateral).

**Table 2:** Bivariate correlations between the studied dimensions of the PLOC, BPNES, and PACES of secondary education students.

Dimensions	M	SD	A	K	1	2	3	4	5	6	7	8	9
1. Intrinsic regulation	5.02	1.43	0.77	0.25	1	0.798 **	0.390 **	−0.124 **	−0.325 **	0.180 **	0.149 **	0.137 **	0.426 **
2. Identified regulation	5.13	1.41	−0.82	0.05		1	0.448 **	−0.051	−0.330 **	0.213 **	0.151 **	0.144 **	0.398 **
3. Introjected regulation	3.93	1.43	−0.10	−0.50			1	0.381 **	0.114 *	0.175 **	0.058	0.180 **	0.151 *
4. External regulation	4.09	1.47	−0.07	−0.64				1	0.372 **	0.027	−0.014	0.040	−0.174 **
5. Amotivation	2.57	1.57	0.90	−0.15					1	−0.049	−0.084	−0.050	−0.258 **
6. Autonomy	3.40	0.88	0.18	0.34						1	0.575 **	0.561 **	0.112 *
7. Competence	3.72	0.85	−0.25	−0.43							1	0.399 **	0.120 *
8. Relatedness	2.95	0.96	0.37	0.65								1	0.094
9. Enjoyment	3.95	0.77	−0.57	−0.68									1

Note: M: mean; SD: standard deviation; A: asymmetry; K: kurtosis; ** The correlation is significant at the 0.001 level (bilateral). * The correlation is significant at the 0.05 level (bilateral).

**Table 3:** Descriptive data of the analyzed variables of the PLOC, BPNES, and PACES. Mean and standard deviation according to gender and educational stage.

		Total		Boys		Girls	
Variable	Educational Stage	M	SD	M	SD	M	SD
Intrinsic regulation (1–7)	Primary	5.83	1.32	5.92	1.14	5.76	1.45
	Secondary	5.02	1.43	5.25	1.26	4.82	1.53
Identified regulation (1–7)	Primary	5.79	1.24	5.89	1.13	5.71	1.32
	Secondary	5.13	1.41	5.32	1.31	4.97	1.47
Introjected regulation (1–7)	Primary	4.25	1.43	4.29	1.33	4.22	1.50
	Secondary	3.93	1.43	4.04	1.46	3.85	1.39
External regulation (1–7)	Primary	3.95	1.71	4.00	1.66	3.91	1.75
	Secondary	4.09	1.47	4.03	1.43	4.13	1.50
Amotivation (1–7)	Primary	2.36	1.56	2.43	1.58	2.30	1.54
	Secondary	2.57	1.57	2.60	1.62	2.55	1.53
Autonomy (1–5)	Primary	3.52	0.90	3.54	0.94	3.49	0.88
	Secondary	3.40	0.88	3.45	0.87	3.37	0.89
Competence (1–5)	Primary	4.13	0.71	4.18	0.70	4.10	0.72
	Secondary	3.72	0.85	3.64	0.87	3.78	0.83
Relatedness (1–5)	Primary	4.40	0.77	4.48	0.70	4.33	0.83
	Secondary	2.95	0.96	3.00	0.95	2.90	0.97
Enjoyment (1–5)	Primary	4.36	0.70	4.43	0.60	4.29	0.77
	Secondary	3.95	0.77	4.03	0.67	3.89	0.83

Note: M: mean; SD: standard deviation.

## Data Availability

The data presented in this study are not available in accordance with Regulation (EU) of the European Parliament and of the Council 2016/679 of 27 April 2016 regarding the protection of natural persons with regard to the processing of personal data and the free circulation of these data (RGPD).

## References

[1] Bandura A. (2018). Toward a Psychology of Human Agency: Pathways and Reflections. Perspect. Psychol. Sci..

[2] Ruiz-Pérez L.M., Moreno-Murcia J.A., Ramón-Otero I., Alias-García A. (2023). Motivación de Logro Para Aprender En Educación Física: Adaptación de La Versión Española del Test AMPET. Rev. Española Pedagog..

[3] González-Gutiérrez I., López-García S., Barcala-Furelos M., Mecías-Calvo M., Navarro-Patón R. (2024). Schoolchildren’s Thinking on the Subject and Teachers of Physical Education According to Gender and Educational Grade. Educ. Sci..

[4] González-Gutiérrez I., López-García S., Barcala-Furelos M., Mecías-Calvo M., Navarro-Patón R. (2025). Self-Perception of Efficacy and Attitudes towards Physical Activity and Sport in Schoolchildren. J. Hum. Sport Exerc..

[5] Bailey R. (2006). Physical Education and Sport in Schools: A Review of Benefits and Outcomes. J. Sch. Health.

[6] Hills A.P., Dengel D.R., Lubans D.R. (2015). Supporting Public Health Priorities: Recommendations for Physical Education and Physical Activity Promotion in Schools. Prog. Cardiovasc. Dis..

[7] González-Cutre D., Sierra A.C., Beltrán-Carrillo V.J., Peláez-Pérez M., Cervelló E. (2018). A School-Based Motivational Intervention to Promote Physical Activity from a Self-Determination Theory Perspective. J. Educ. Res..

[8] Guan J., Xiang P., Land W.M., Hamilton X.D. (2023). The Roles of Perceived Physical Education Competence, Enjoyment, and Persistence on Middle School Students’ Physical Activity Engagement. Percept. Mot. Ski..

[9] Teixeira P.J., Carraça E.V., Markland D., Silva M.N., Ryan R.M. (2012). Exercise, Physical Activity, and Self-Determination Theory: A Systematic Review. Int. J. Behav. Nutr. Phys. Act..

[10] Vasconcellos D., Parker P.D., Hilland T., Cinelli R., Owen K.B., Kapsal N., Lee J., Antczak D., Ntoumanis N., Ryan R.M. (2020). Self-Determination Theory Applied to Physical Education: A Systematic Review and Meta-Analysis. J. Educ. Psychol..

[11] Ntoumanis N., Quested E., Reeve J., Cheon S.H. (2017). Need Supportive Communication: Implications for Motivation in Sport, Exercise, and Physical Activity. Persuasion and Communication in Sport, Exercise, and Physical Activity.

[12] Castillo I., Molina-García J., Estevan I., Queralt A., Álvarez O. (2020). Transformational Teaching in Physical Education and Students’ Leisure-Time Physical Activity: The Mediating Role of Learning Climate, Passion and Self-Determined Motivation. Int. J. Environ. Res. Public Health.

[13] Ryan R.M., Deci E.L. (2017). Self-Determination Theory: Basic Psychological Needs in Motivation, Development, and Wellness.

[14] Ryan R.M., Deci E.L. (2020). Intrinsic and Extrinsic Motivation from a Self-Determination Theory Perspective: Definitions, Theory, Practices, and Future Directions. Contemp. Educ. Psychol..

[15] Vansteenkiste M., Ryan R.M., Soenens B. (2020). Basic Psychological Need Theory: Advancements, Critical Themes, and Future Directions. Motiv. Emot..

[16] Baumeister R.F., Leary M.R. (2017). The Need to Belong: Desire for Interpersonal Attachments as a Fundamental Human Motivation. Interpersonal Development.

[17] Fernández-Hernández A., Manzano-Sánchez D., Jiménez-Parra J.F., Valero-Valenzuela A. (2021). Analysis of Differences According to Gender in the Level of Physical Activity, Motivation, Psychological Needs and Responsibility in Primary Education. Proceedings of the Journal of Human Sport and Exercise Autumn Conferences of Sports Science.

[18] Howard J., Gagné M., Morin A.J.S., Van den Broeck A. (2016). Motivation Profiles at Work: A Self-Determination Theory Approach. J. Vocat. Behav..

[19] Gagné M., Deci E.L., Ryan R.M. (2018). Self-Determination Theory Applied to Work Motivation and Organizational Behavior. The SAGE Handbook of Industrial, Work & Organizational Psychology.

[20] Van den Broeck A., Ferris D.L., Chang C.-H., Rosen C.C. (2016). A Review of Self-Determination Theory’s Basic Psychological Needs at Work. J. Manag..

[21] Howard J.L., Gagné M., Bureau J.S. (2017). Testing a Continuum Structure of Self-Determined Motivation: A Meta-Analysis. Psychol. Bull..

[22] Miquelon P., Castonguay A. (2017). Integrated Regulation, Behavior Consistency, and Physical Activity Maintenance. Motiv. Sci..

[23] Teixeira D.S., Bastos V., Andrade A.J., Palmeira A.L., Ekkekakis P. (2024). Individualized Pleasure-Oriented Exercise Sessions, Exercise Frequency, and Affective Outcomes: A Pragmatic Randomized Controlled Trial. Int. J. Behav. Nutr. Phys. Act..

[24] Navarro-Patón R., Lago-Ballesteros J., Arufe-Giráldez V. (2020). Midiendo La Motivación Auto-Determinada Hacia La Educación Física En La Escolaridad Obligatoria. J. Sport Psychol..

[25] Arsani A., Maksum A., SyamTuasikal A.R., Kusnanik N.W. (2020). Differences in Motivational Orientation in Physical Education in Terms of Gender Differences. Bp. Int. Res. Crit. Linguist. Educ. J..

[26] Erdvik I.B., Haugen T., Ivarsson A., Säfvenbom R. (2019). Development of Basic Psychological Need Satisfaction in Physical Education. J. Res. Arts Sports Educ..

[27] Wang L., Chen R. (2022). Psychological Needs Satisfaction, Self-determined Motivation, and Physical Activity of Students in Physical Education: Comparison across Gender and School Levels. Eur. J. Sport Sci..

[28] Navarro-Patón R., Lago-Ballesteros J., Basanta-Camiño S., Arufe-Giraldez V. (2019). Relation between Motivation and Enjoyment in Physical Education Classes in Children from 10 to 12 Years Old. J. Hum. Sport Exerc..

[29] Navarro-Patón R., Lago-Ballesteros J., Basanta-Camiño S., Arufe Giráldez V. (2018). Assessment of the Basic Psychological Needs in Physical Education According to Age, Gender and Educational Stage. J. Hum. Sport Exerc..

[30] Ato M., López-García J.J., Benavente A. (2013). Un Sistema de Clasificación de Los Diseños de Investigación En Psicología. Anal. Psicol..

[31] Murcia J.A.M., Coll D.G.-C., Garzón M.C. (2009). Preliminary Validation in Spanish of a Scale Designed to Measure Motivation in Physical Education Classes: The Perceived Locus of Causality (PLOC) Scale. Span. J. Psychol..

[32] Goudas M., Biddle S., Fox K. (1994). Perceived Locus of Causality, Goal Orientations, and Perceived Competence in School Physical Education Classes. Br. J. Educ. Psychol..

[33] Vlachopoulos S.P., Michailidou S. (2006). Development and Initial Validation of a Measure of Autonomy, Competence, and Relatedness in Exercise: The Basic Psychological Needs in Exercise Scale. Meas. Phys. Educ. Exerc. Sci..

[34] Menéndez Santurio J.I., Fernández-Río J. (2018). Versión Española de la Escala de Necesidades Psicológicas Básicas en Educación Física/Spanish Version of the Basic Psychological Needs In Physical Education Scale. Rev. Int. Med. Cienc. Act. Física Deport..

[35] Motl R.W., Dishman R.K., Saunders R., Dowda M., Felton G., Pate R.R. (2001). Measuring Enjoyment of Physical Activity in Adolescent Girls. Am. J. Prev. Med..

[36] Moreno J.-A., González-Cutre D., Martínez C., Alonso N., López M. (2008). Propiedades Psicométricas de la Physical Activity Enjoyment Scale (PACES) en el Contexto Español. Estud. Psicol..

[37] Cohen J. (2013). Statistical Power Analysis for the Behavioral Sciences.

[38] World Medical Association (2013). World Medical Association Declaration of Helsinki. J. Am. Med. Assoc..

[39] Jibaja-Barreda A. (2022). Estilo Motivacional Docente, Necesidades Psicológicas Básicas y Metacognición En Matemática En Niños de Primaria. Escr. Psicol. Psychol. Writ..

[40] Rodríguez Torres Á.F., Rodríguez Alvear J.C., Guerrero Gallardo H.I., Arias Moreno E.R., Paredes Alvear A.E., Chávez Vaca V.A. (2020). Beneficios de La Actividad Física Para Niños y Adolescentes En El Contexto Escolar. Rev. Cuba. Med. Gen. Integral.

[41] Durán Fonseca T.D.D., Acle Tomasini G. (2022). Escala de Motivación Escolar Para Alumnos de Primaria: Evidencias de Validez y Confiabilidad. Estud. Pedagógicos.

[42] Luque Illanes A., Gálvez Casas A., Gómez Escribano L., Escámez Baños J.C., Tárraga Marcos L., Tárraga López P.J. (2021). ¿Mejora La Actividad Física El Rendimiento Académico En Escolares?. Una Revisión Bibliográfica J. Negat. No Posit. Results.

[43] Lara Nieto-Márquez N., Garcia-Sinausia S., Pérez Nieto M.Á. (2021). Relaciones de la Motivación con la Metacognición y el Desempeño en el Rendimiento Cognitivo en Estudiantes de Educación Primaria. Anal. Psicol..

[44] Gutiérrez D., García-López L.M., Hastie P.A., Segovia Y. (2022). Adoption and Fidelity of Sport Education in Spanish Schools. Eur. Phys. Educ. Rev..

[45] Fitton Davies K. (2021). An Exploration of Young Children’s Motivational Processes within Physical Education. Ph.D. Thesis.

[46] Lohbeck A., Hohmann A., von Keitz P., Daseking M. (2022). Children’s Motivation Profiles in Sports and Physical Activities: A Latent Profile Analysis and Self-Determination Theory Approach. J. Sport. Exerc. Psychol..

[47] Manzano-Sánchez D. (2023). Profile Analysis through Self-Determination Theory and Intention to Be Physically Active: Differences According to Gender and Age. Front. Psychol..

[48] Fierro-Suero S., Castillo I., Almagro B.J., Saénz-López P. (2023). The Role of Motivation and Emotions in Physical Education: Understanding Academic Achievement and the Intention to Be Physically Active. Front. Psychol..

[49] Jaitner D., Rinas R., Becker C., Niermann C., Breithecker J., Mess F. (2019). Supporting Subject Justification by Educational Psychology: A Systematic Review of Achievement Goal Motivation in School Physical Education. Front. Educ..

[50] Paulson L., Knipe R. (2023). Physical Activity Used as Punishment: Advocating for Better Practices. J. Phys. Educ. Recreat. Danc..

[51] Portela-Pino I., López-Castedo A., Martínez-Patiño M.J., Valverde-Esteve T., Domínguez-Alonso J. (2019). Gender Differences in Motivation and Barriers for The Practice of Physical Exercise in Adolescence. Int. J. Environ. Res. Public Health.

[52] Rojo-Ramos J., Franco-García J.M., Mayordomo-Pinilla N., Pazzi F., Galán-Arroyo C. (2023). Physical Activity and Emotional Regulation in Physical Education in Children Aged 12–14 Years and Its Relation with Practice Motives. Healthcare.

[53] Abós Á., Burgueño R., García-González L., Sevil-Serrano J. (2022). Influence of Internal and External Controlling Teaching Behaviors on Students’ Motivational Outcomes in Physical Education: Is There a Gender Difference?. J. Teach. Phys. Educ..

[54] Frikha M., Chaâri N., Mezghanni N., Hassan A.K., Alhumaid M.M., Alibrahim M.S. (2024). Enhancing Institutional Integration and Enjoyment among Saudi Female Physical Education Students: Exploring the Mediation of Motivation and Psychological Needs Satisfaction. Front. Educ..

[55] Brisson R., Mendes F.G., Catunda C. (2023). Accounting for the Gender Gap in Adolescents’ Life Satisfaction: Evidence from Nationally Representative Samples of School Attendees in Luxembourg. Int. J. Adolesc. Youth.

[56] Monzó M.P., García Martínez S., Jaén M.G., Valero A.F. (2021). Orientaciones de Meta y Necesidades Psicológicas Básicas en el desarrollo de la expresión corporal en Educación Primaria: Un Estudio Piloto (Goal Orientations and Basic Psychological Needs in the Development of Corporal Expression in Primary Education: A Pilot Study. Retos.

[57] Cachón-Zagalaz J., Carrasco-Venturelli H., Sánchez-Zafra M., Zagalaz-Sánchez M.L. (2023). Motivation toward Physical Activity and Healthy Habits of Adolescents: A Systematic Review. Children.

[58] Hortigüela-Alcalá D., Barba-Martín R.A., González-Calvo G., Hernando-Garijo A. (2021). ‘I Hate Physical Education’; an Analysis of Girls’ Experiences throughout Their School Life. J. Gend. Stud..

